# Mechanical Response of Epoxy Resin—Flax Fiber Composites Subjected to Repeated Loading and Creep Recovery Tests

**DOI:** 10.3390/polym15030766

**Published:** 2023-02-02

**Authors:** Constantin Stochioiu, Anton Hadăr, Benoît Piezel

**Affiliations:** 1 Department of Strength of Materials, Faculty of Industrial Engineering and Robotics, University Politehnica of Bucharest, 313 Splaiul Independenței, 060042 Bucharest, Romania; 2 Academy of Romanian Scientits, 3 Ilfov Street, 050045 Bucharest, Romania; 3 Technical Sciences Academy of Romania, 26 Dacia Boulevard, 030167 Bucharest, Romania; 4 DRIVE Lab, University of Burgundy, F-58000 Nevers, France

**Keywords:** fiber-reinforced plastics, viscoelasticity, viscoplasticity, mechanical characterization

## Abstract

Flax fiber-reinforced plastics have an innate eco-friendly nature due to the fiber reinforcement and reduced energy requirements in fabrication when compared to current fiber reinforced composite materials. They possess a complex time-dependent material behavior, which is investigated in the present paper. A composite material with flax fiber reinforcement on the load direction, embedded in an epoxy resin matrix, was studied. The procedures used were tensile tests, repeated loading-recovery, and creep-recovery tests, which were meant to expose the components of the response with respect to stress level and load duration. The results showed an elastic bi-linear behavior, a yield point at approximately 20% of the ultimate tensile stress, and tensile moduli of 35.9 GPa and 26.3 GPa, before and after yield. This is coupled with significant non-linear viscoelastic and, after yield, viscoplastic components, accounting for up to 14% of the strain response. The behavior is inherited from both the matrix and the fiber reinforcement and is attributed to the amorphous nature of the matrix combined with the microstructural re-organization of the fiber under load, which are partially reversible.

## 1. Introduction

The application range for fiber reinforced plastics (FRPs) has seen an increase in their use as structural components in various areas of industry. Component quality has been shown to improve when incorporating FRPs in areas such as fluid transfer [1,2], transportation [3], and civil construction [4]. Their high strength and stiffness, combined with low density and resistance to corrosion, lead to light weight components, which are suitable for utilization in harsh conditions [1,2,3,5,6]. Their main drawback is the amount of pollution associated with their exploitation. The amount of energy required for fabrication is high, and the solutions for the component′s end of life cycle are unsatisfactory, leading to high amounts of non-biodegradable waste [7]. 

Plant fiber reinforced plastic materials are taken into consideration as a possible solution for these issues [8,9,10]. Their nature makes them an eco-friendly and a bio-degradable solution. From the studied plant reinforcements, flax fiber has been shown to be the most prominent. It possesses the highest mechanical properties [9,11,12,13,14], competitive vibration damping [15,16], and energy absorption capabilities [14,17] when compared to glass or carbon fiber composites, as well as a low density, which leads to high specific strength and stiffness [18].

It is being studied for parallel adoption, either for hybridization [19,20,21], or as a replacement for conventional materials in several sectors, such as automotive [22,23,24], energy [25], and construction [26]. The results, in most cases, showed improvement of the overall quality of components containing flax fiber reinforced plastics (FFRPs) when compared to the initial material, in terms of ecological impact, energy requirements for production, and mass reduction [22]. The encouraging results fuel the development of materials with flax fiber reinforcement, especially in areas with a temperate climate, where flax can be cultivated locally [18].

Despite the advantages of FFRPs, they are still used in a limited number of structural applications. This is mainly due to the mechanical and hygrothermal behavior of the resulting composite under various loads and environmental conditions, which are yet to be fully understood [27,28]. 

The mechanical behavior of the flax fiber is complex, with tensile tests showing a bilinear and even a trilinear evolution [18,29,30,31], with a yield point at relatively low stress levels [32]. The mechanisms involved are connected to the complex structure of the fiber. The main components are cellulose microfibrils, which provide load bearing capacity, and an amorphous hemicellulose matrix in which they are embedded. The microfibrils are organized in a helicoidal configuration along the fiber length. When loaded, the microstructure tends to reorganize, resulting in a viscous response in the amorphous phase and an alignment of the microfibrils to the load direction [30,33]. The viscous component of the fiber behavior has been demonstrated by Keryvin et. al. through nano indentation relaxation tests [31] and by Charlet through repeated loading tests [34]. This, along with the innate viscous behavior of a plastic matrix, such as epoxy [35], produce a composite which inherits a similar response. The mechanisms leading to a viscous response are related to the amorphous polymer chains motion of both the composite and the fiber matrix in the direction of stress, when specific activation energies are reached [36,37]. The matrix reorganization leads to a continuous variation of strain and a reduction in stiffness. 

The viscous behavior has been documented in the literature for FFRPs reinforced with short fibers [38], weavings [39], and long fibers along the load direction [40]. The behavior of the former two configurations has been associated with viscoelasticity and viscoplasticy. For the latter configuration, the time-dependent behavior is a novelty, as similar composites containing synthetic fibers lack such a response [41] due to the dominant behavior of the reinforcement, which is purely linear elastic. 

The viscous response has been used to explain the bi-linear evolution of the stress–strain curve of FFRPs, considering that it becomes important after the yield point. This has been exposed with variable rate tensile tests by Poilâne et al. [42] and Giuliani P.M. et. al. [43]. They showed a stiffening of the material with an increasing strain rate. Additionally, repeated loading tests showed plastic strains, hysteresis loops, and a similar bi-linear evolution during loading, with a shifting of the yield point [42,44]. However, as shown by Pitarresi [44], the yield point shift tends to be eliminated by adding a recovery period between loadings. These results reinforce the idea of viscous reversible and irreversible strains, which appear after yield.

Creep-recovery tests performed on unidirectional FFRPs show a variable deformation during both phases and irreversible strains at the end of recovery. Sala et al. [28] noted that both the reversible and remanent strains depend on stress level. The results suggested a linear viscoelastic strain, with respect to stress level, and independence of plastic deformations, with respect to time [28,42,45]. 

However, on similar plant reinforced materials, variable creep duration tests showed that the viscoelasticity is non-linear, and plastic strains varied with time [46]. As an additional behavior, Marklund et al. [47] observed that in composites incorporating hemp, a similar fiber, there is a possible altering of mechanical properties at high stress levels due to damage.

The information available in the literature regarding similar plant reinforced composites has led to speculate that FFRPs should also exhibit a non-linear viscoelastic and viscoplastic strain response under load. Thus, the mechanical behavior has been studied in the present work, with the intent of addressing these unknowns. The time-dependent components of the behavior were emphasized through a series of experimental procedures which encompassed tensile tests, repeated loading-unloading tests, and creep-recovery on samples with no loading history and with mechanical conditioning. The tested material was a flax fiber–epoxy resin composite, with fiber reinforcement on the direction of load. Additionally, the repeated loading of the tested samples permitted an analysis of any potential degradation of mechanical properties.

It is worth noting that, in the present work, viscous is a general term which refers to time-dependent behavior. More appropriately, the term viscoelastoplastic was adopted for the next sections to describe that the material response contains both a reversible component, which is viscoelastic and an irreversible, as well as time dependent element, which is viscoplastic.

## 2. Materials and Methods

### 2.1. Material Fabrication

The studied material was a unidirectional flax fiber–epoxy resin composite, commercially known as FlaxPreg T-UD 110, produced by EcoTechnilin© (Valliquerville, France) [48]. It was supplied as a ready to use, pre-impregnated roll, with a fiber density of 110 g/m^2^ and a 50%-50% fiber/resin mass ratio.

From the roll, 15 layers were cut, 280 mm × 280 mm in size, and stacked on the same fiber direction in a mold, for a desired end thickness of 2 mm. A thermocompression cycle was applied using a Frontijne Grotness TPC heated press (Delft, The Netherlands). The cycle was based on the FlaxPreg T-UD 110 technical data sheet and the work of Cadu et al. [49]. It consisted of an increase in temperature of 5 °C/min up to 130 °C, which was maintained for 60 min, and a pressure of 3 bars starting at 115 °C.

The cooling phase of the cycle was achieved with a temperature decrease of 2 °C/min to ensure uniform cooling and the absence of temperature related residual stress. Finally, the composite plates were subjected to a post-curing cycle of 130 °C for 1 h to ensure complete resin reticulation.

### 2.2. Sample Preparation

The fabricated plates were laser cut into strips with a rectangular geometry of 250 mm × 25 mm. A total of 13 samples were subjected to testing procedures, as is detailed in the following sections.

**Figure 1:**
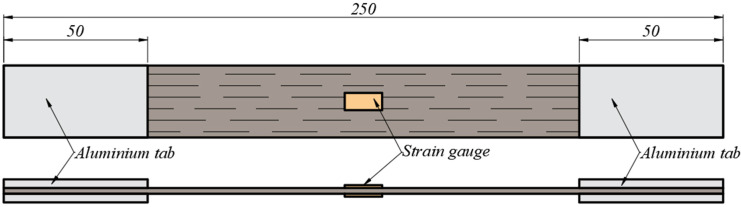
Sample equipped with tabs and strain gauges.

Aluminum tabs were glued on the samples to eliminate possible stress concentrations caused by fixture in the testing equipment. The specimens to be analyzed were equipped with strain gauges for deformation measuring. They were mounted in a full Wheatstone bridge, with one on each face of the sample (Figure 1) and two dummy gauges. This configuration allows for temperature compensation and the elimination of flexion-induced strains.

Prior to testing, the samples were conditioned for seven days in a controlled environment, with 23 ± 3 °C and 50 ± 5% humidity.

### 2.3. Experimental Procedures

**Figure 2:**
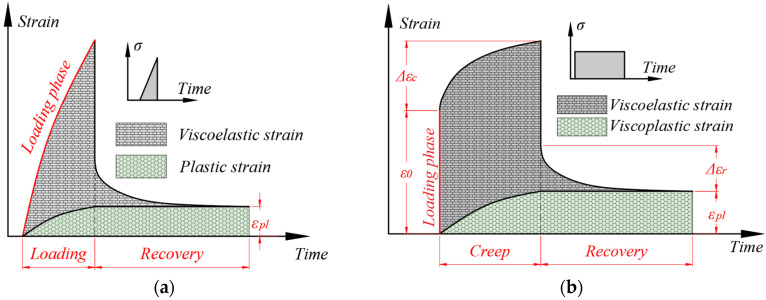
Schema of the expected strain response: (**a**) during a loading-recovery test; (**b**) during a creep-recovery test.

Several types of tests were conducted on a total of 13 samples with the purpose of distinguishing the components of the material′s response. They consisted of several types of loading-recovery tests and creep-recovery tests, for which the expected strain responses are shown in Figure 2. The procedures were as follows:

- Tensile testing, to determine the quasi-static mechanical properties, was conducted on five samples.
- Repeated progressive load (RPL) test, to determine the effects of stress level on the mechanical response, were performed on five samples.
- Repeated regressive loading speed (RRLS) test, conducted to distinguish any time-dependent components of the mechanical behavior during loading, was performed on one sample.
- Cyclic creep-recovery test with regressive load, which were designed to identify the influence of stress on the time-dependent behavior, was conducted on one sample.
- Cyclic creep-recovery test with progressive creep duration, which was used to identify the influence of load duration on the viscous behavior, was performed on one sample.

For a loading-recovery test (Figure 2a), during the loading phase, a coupling of elastic and viscoelastic strains was expected, with possible plastic strains appearing at high loads. When the load is removed, the elastic component is eliminated instantaneously and, during recovery, the viscoelastic strain disperses, allowing, at the end of the phase, for the distinguishing of any plastic strain, ε_pl_, which could only have accumulated during loading.

For a creep-recovery test (Figure 2b), during loading, an elastic response (ε_0_) was expected. The impossibility of instantaneous loading leads to the appearance of a viscous component as well. However, it is significantly lower than the one produced during the creep period, and for the present work, it was neglected. Creep produces a total transient strain, (Δε_c_), and recovery generates a lower transient strain (Δε_r_), which is purely viscoelastic. At the end of the cycle, the non-recovered strain, denoted as ε_pl_, is measured. The loading domain was selected to avoid possible stress-induced damage, which could have disturbed the experimental results.

Considering the expected strain response, the results of tensile and RPL tests formed the basis for the choice of parameters in the subsequent procedures, as follows:

- The yield point coordinates allowed for the choice of stress levels for the multi-cycled procedures.
- The formation of plastic strain prompted its elimination in certain procedures through mechanical conditioning.
- The analysis of the effects of progressive loading allowed for the use of the samples for multiple loading cycles with stress levels superior to the yield point.

#### 2.3.1. Tensile Tests

The quasi-static material properties of the material in the longitudinal direction were determined through tensile tests. The machine used was an INSTRON 8872 (Norwood, MA, USA) electro-hydraulic Universal Testing Machine (UTM) equipped with a 25 kN load cell. Tests were conducted in accordance with ASTM D3039, with a crosshead speed of 2 mm/min, and the strain recording was obtained with a frequency of 10 Hz by an HBM QuantumX (Darmstadt, Germany) data acquisition system. One of the samples was equipped with an additional set of strain gauges mounted perpendicular to the load direction, which allowed for the extraction of Poisson′s ratio.

#### 2.3.2. Repeated Progressive Load (RPL) Tests

Plastic strains due to loading were analyzed through multi-cycled loading-recovery tests, with increasing stress levels. Since the use of other plant-based reinforced materials has shown that high stress levels risked altering the material′s behavior, the possible effects of damage were also evaluated.

Each cycle consisted of several phases:

1. Loading up to the desired stress level.
2. Unloading by opening one of the machine′s grips.
3. Recovery for several minutes to reduce viscoelastic strain.
4. A low stress loading-unloading phase, destined to evaluate tensile modulus degradation with respect to stress level.

The loading speed was chosen as 50 kN/min (approximately 10 mm/min, considering sample dimensions) to reduce the amount of viscoelastic deformation during loading. For phases with stress levels nearing failure and the evaluation phases, the speed was reduced to 10 kN/min (approx. 2 mm/min) for comparison with the tensile tests. The recovery phases lasted until complete strain stabilization (for lower stress levels) or until reaching a strain variation lower than 10 µm/m during one minute of recording.

**Figure 3:**
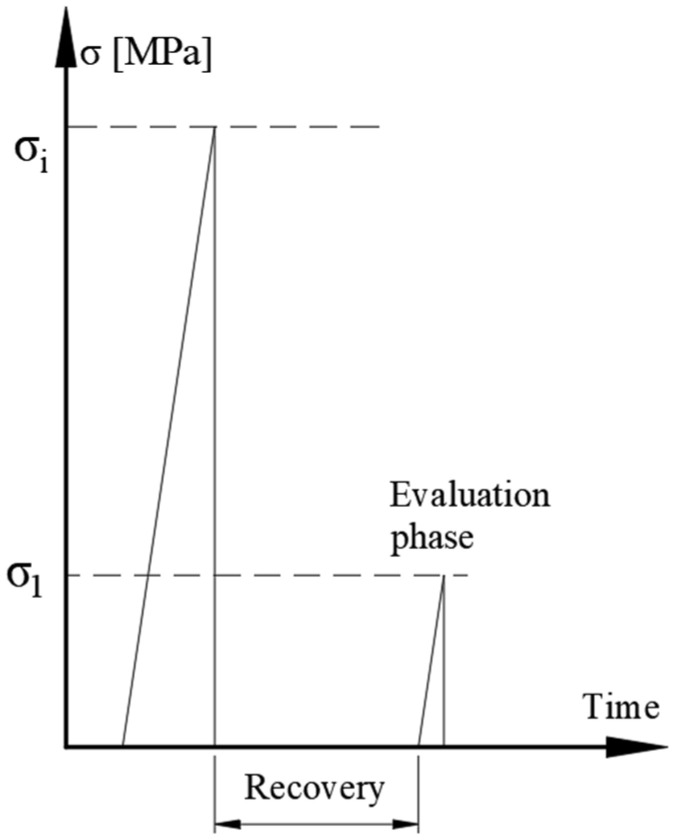
Load cycle with evaluation phase.

The testing procedure and the stress levels are presented in Figure 3 and Table 1. The stress level for the evaluation phases was 30 MPa, in the first region of the stress–strain curve. Therefore, the first load cycle, which has the same stress level, does not require an additional evaluation phase.

Sample loading was made with the Instron 8872 UTM, and strain measurements were conducted with strain-gauges, at a frequency of 50 Hz.

#### 2.3.3. Repeated Regressive Loading Speed (RRLS) Tests

The viscous component of the behavior during loading was analyzed by varying the speed during repeated loading tests, while loading to the same stress level. The procedure and equipment were similar to those presented in the previous section. There were two notable distinctions: the sample was mechanically conditioned prior to testing, and the evaluation cycles were eliminated, as they would provide no useful information due to the conditioning. The cycle parameters are presented in Table 2.

Load speeds were selected in multiple orders of magnitude, with the highest being limited to avoid the appearance of shock and load overshoot. The stress level was chosen in the second portion of the stress–strain curve, superior to the yield stress. The mechanical conditioning was conducted at a higher load level to eliminate possible plastic strains. The strain response recorded would, therefore, only be viscoelastic.

Due to higher loading speeds, the sampling frequency has been increased to 300 Hz.

#### 2.3.4. Cyclic Creep-Recovery with Regressive Load

**Figure 4:**
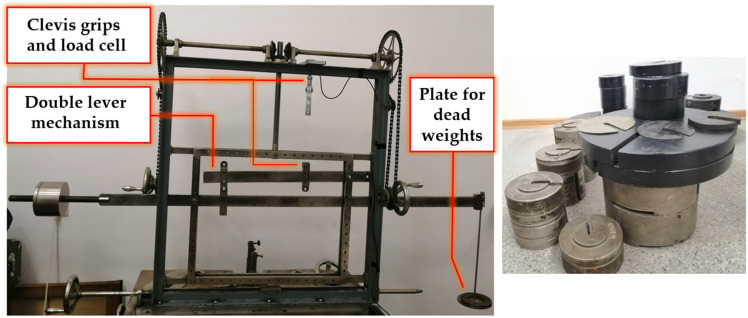
The installation used for creep-recovery testing.

For further analysis of only the viscoelastic component, the long-term effect of stress was analyzed through a series of repeated creep-recovery tests with various creep stress levels. Mechanical conditioning was introduced to eliminate plastic strains in further testing cycles, ensuring the measurement of only viscoelastic strains. Loading was achieved with a double-lever mechanism and dead weights (Figure 4). The effective load was measured with a HBM U9B (Darmstadt, Germany) 20 kN load cell mounted in series with the sample in the testing equipment and connected to the QuantumX data acquisition system. This allowed for real-time recording of stress, by dividing the measured force to the sample’s cross-sectional area. Strain measurement was performed with the same strain gauge configuration previously presented, at a sample rate of 2 Hz, to allow for recording during long periods of time. Between the creep cycles, a recovery period was introduced to allow strain stabilization, which was considered complete when a variation of less than 10 µm/m was recorded for ten consecutive hours.

The tested samples are drilled in the tab region to allow mounting in the installation’s clevis fasteners. This method ensures that the sample is solely axially loaded and that no sliding occurs in the fixtures.

The mechanical conditioning was achieved with a creep-recovery cycle at 95 Mpa for 3 h to ensure the elimination of plastic deformation during the subsequent creep cycles. The conditioning cycle parameters were selected to reduce the risks of sample damage or failure during testing.

**Figure 5:**
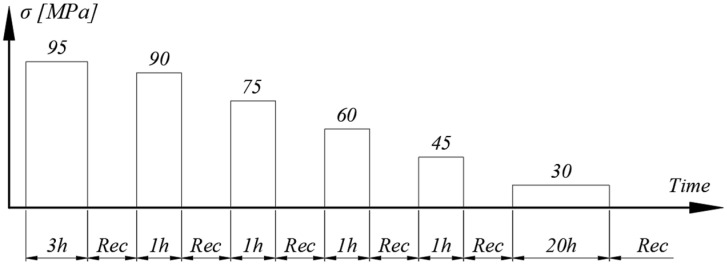
Creep-recovery with variable creep stress.

The testing cycle is represented as a diagram in Figure 5, where “Rec” denotes the recovery period.

The lowest stress level was in the first portion of the stress–strain curve, in which the viscous response is considered low. Consequently, to ensure viable viscoelastic strain reading, the creep period was extended to 20 h.

#### 2.3.5. Cyclic Creep-Recovery with Progressive Creep Duration

**Figure 6:**
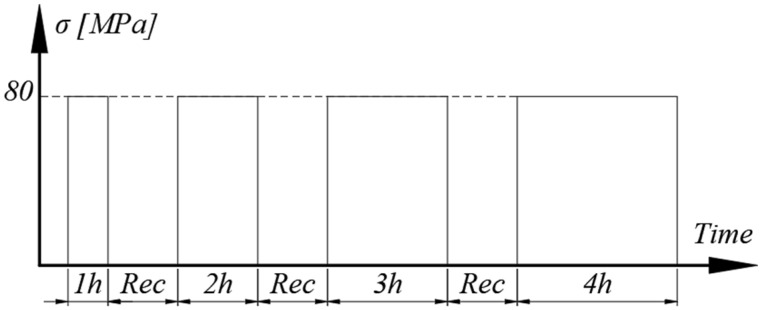
Creep-recovery with variable creep duration.

This test aimed at verifying the existence of a viscoplastic response. An experimental procedure was designed, consisting of four creep phases, followed by recovery. The creep stress was the same for all phases and the duration increased from one to four hours. The testing cycle is represented in Figure 6, where “Rec” denotes the recovery period. The stress level was chosen to ensure that only first-stage creep appeared while accumulating, if present, viscoplastic strains, while also reducing the risk of damage on the sample.

Since recovery is a phase with no load, the plastic strain remains constant, and the value extracted at the end is only a product of creep. In terms of viscoplasticity, recovery acts as an interruption, and creep as a continuation of strain accumulation. Thus, viscoplastic deformation extracted at the end of a given recovery phase is the result of the cumulated creep duration. By using the same stress level for creep, any variation of plastic strain would be creep duration dependent, denoting the existence of the viscoplastic behavior.

The testing and measurement equipment, along with the strain sampling rate, are the same as for the test presented in the previous section. The expected result follows the same scheme of Figure 2b, with the notable exception that, if time-dependent plastic strains appear, *ε_pl_* varies from one cycle to the next.

## 3. Results and Discussion

The presented results are grouped and discussed, considering the following aspects:

- The type of mechanical response in quasi static tensile load along with the material’s mechanical properties.
- The components of strain response with respect to load and the possible effects of sample damage.
- The effects of a viscoelastic component during loading and when the load is maintained.
- The quantification of possible viscoplastic effects with respect to load duration.

The specifics relating to the testing procedure, parameters, and experimental outcomes are discussed. The type of behavior of the flax fiber–epoxy resin composites regarding load, load level, and load duration is to be inferred, as well as, if present, any thresholds for the activation of specific behaviors.

### 3.1. Quasi-Static Mechanical Properties

**Figure 7:**
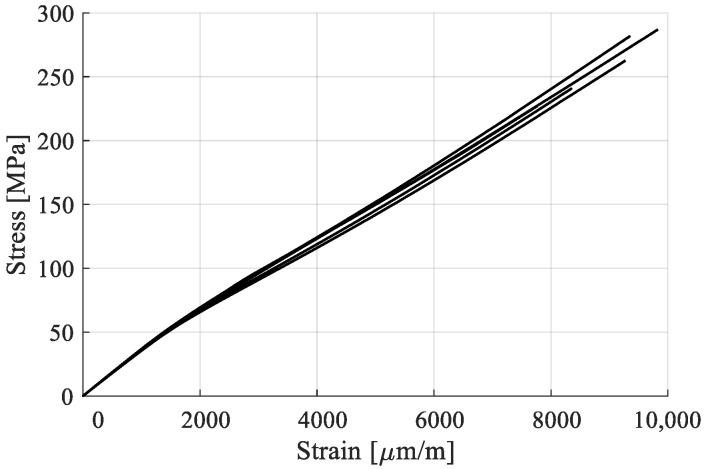
Stress–strain curves of flax fiber—epoxy resin composites.

**Figure 8:**
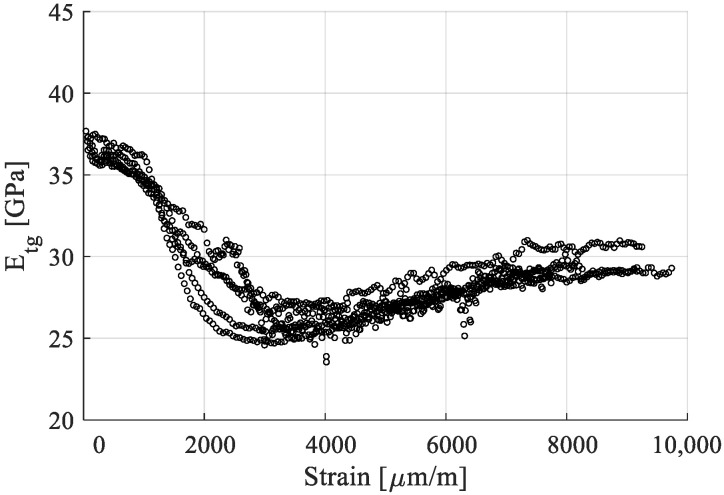
Tangent modulus with respect to strain.

The stress–strain curves obtained for the five samples are presented in Figure 7. A nonlinear evolution is noticeable. The phenomenon is better observed when plotting the evolution of tangent modulus with respect to the strain, as shown in Figure 8. Since, on this direction of the reinforcement, properties are fiber dominated, the stress–strain curve is similar to that of the fibers.

The tangent modulus varies continuously, with notable changes between 1000 and 2000 µm/m strain, when the modulus falls from 35 to 40 Gpa to about 25 to 30 Gpa. This result led to considering modeling the evolution as bilinear in several works [32,50], with Poilâne proposing a supplementary stiffening factor for the second half of the curve, where the tangent modulus has a slight increase [51].

**Figure 9:**
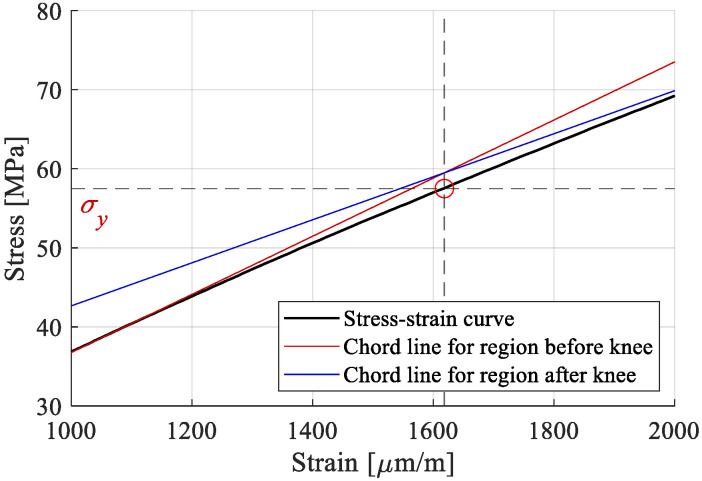
Detail on the calculus of $\sigma_{y}$.

For the present work, a bilinear evolution was chosen, with a yield stress at the “knee” region, $\sigma_{y}$, and tensile modulus for the portion before the knee, E_1_, and one after, E_2_. E_1_ and E_2_ were calculated as the tangent moduli regions 100–1000 µm/m and 3000–5000 µm/m, respectively. Both were obtained by linear regression for their respective portion of the stress–strain curve. $\sigma_{y}$ was determined as the corresponding stress coordinate of the strain intersection of the two chords that approximate the curves (Figure 9). The ultimate tensile stress, $\sigma_{u}$*,* and ultimate strain, $\varepsilon_{u}$, were extracted as the coordinates of the curve at the sample failure. The calculated values, extracted as means from the stress–strain curves, the standard deviations, and the coefficients of variation are presented in Table 3.

The yield stress, $\sigma_{y}$, was obtained at a rather low value, approximately 20% of the ultimate tensile stress. A tensile modulus of 35.88 Gpa was calculated for the first region, comparable to the one obtained by the producer (35 Gpa [48]). A decrease in the modulus of 26.7% has been recorded between the two regions of the stress–strain curve.

### 3.2. Tensile Behavior

**Figure 10:**
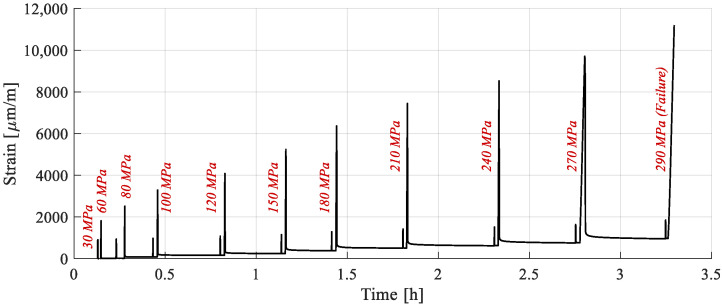
Strain response of a sample subjected to repeated progressive load tests.

The strain response of one of the samples subjected to RPL tests is presented in Figure 10. As expected, a time varying strain response is present, highlighted by strain variation during the recovery phases of all cycles. Beginning with the 60 Mpa cycle, the sample does not fully recover, indicating the onset of plastic strain accumulation with increasing load level until failure.

**Figure 11:**
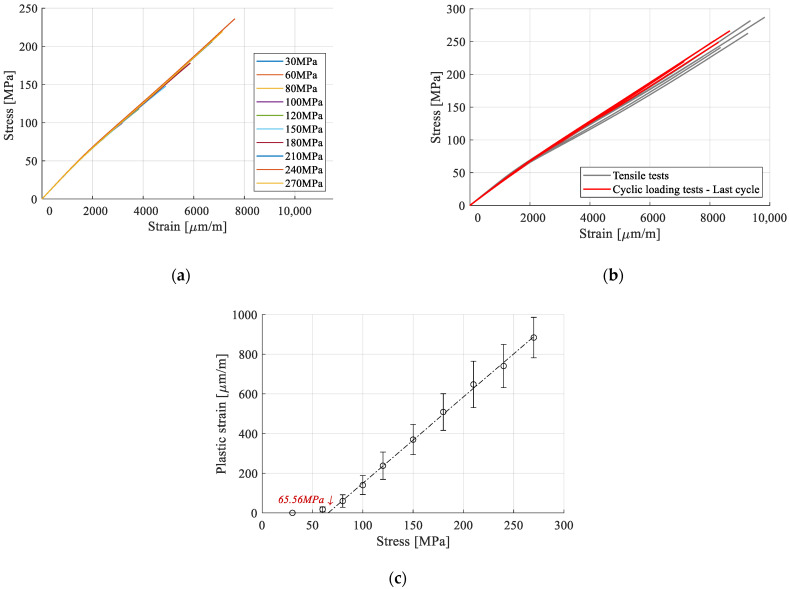
Analysis of the strain response: (**a**) loading phases in stress–strain coordinates; (**b**) comparison of stress–strain curves between fresh samples and those subjected to multiple stress levels; (**c**) plastic strain with respect to cycle stress level.

According to acoustic emission experiments on FFRPs published in the literature, composites with fibers oriented in the direction of load suffer significant damage in the sample, starting at about 40% of the load level. This indicates that modulus loss owing to damage should be expected. However, the loading phases of the representative sample, extracted from Figure 10, and analyzed in Figure 11a, are superposed, indicating that no alteration of stiffness has occurred. This result is corroborated with the evaluation cycles in which E_1_ was calculated, for all cycles to a value of 35.75 ± 0.15 Gpa, with no noticeable variations. This indicated that the minor damage which appears (fiber and matrix rupture, debonding) does not have a significant influence on the material’s mechanical properties.

The final loading cycle for each sample is presented next to the tensile tests results in Figure 11b. Accumulated plastic strain has been subtracted for comparison purposes. Between the two sets, there are no appreciable differences, supporting the finding that damage has not changed the mechanical properties. A slight increase in stiffness after yield can be observed, which is attributed to the fact that the plastic component is mostly accumulated in the cycles preceding failure, leading to a lower amount in the last cycle.

Since damage is certain, yet the experimental findings revealed the appearance of plastic deformation and no loss of modulus, it may be assumed that the effects of damage are on the material′s permanent deformation and not on its mechanical characteristics. Furthermore, neither plastic strains nor damage modify the general bilinear evolution of the stress–strain curve, leading to the conclusion that the “knee” region is stress, and not strain, dependent. The yield point does not shift after repeated loading if recovery is introduced, which is consistent with the findings of Pitarresi [44].

Finally, the plastic strains accumulated at the end of each loading cycle are extracted for all samples (Figure 11c). They follow a linear evolution with respect to stress, allowing for the extrapolation of the stress level at which plastic strains start forming. It has been calculated at 65 MPa, close to the yield stress of 58 MPa determined in the tensile tests, indicating that the two thresholds are superposed. The result is coherent with work available in the literature, where plastic strain accumulation has been observed after the yield stress [52,53].

The resulting experimental data is presented in Table 4 by means of E_1_, E_2_, and plastic strain, as average and standard deviation for the conducted tests. In the case of the tensile moduli, the intervals for calculation are kept the same as those for the mechanical characterization tests. Consequently, *E*_2_ was only calculated for load levels that produce a strain response higher than 5000 µm/m.

**Figure 12:**
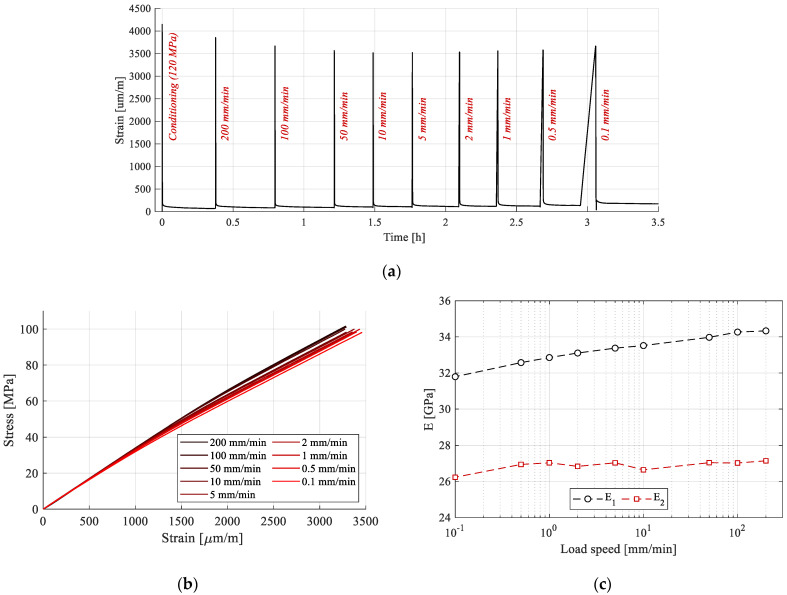
Analysis of the strain response during loading-recovery tests: (**a**) response during the testing procedure; (**b**) stress–strain curves for variable load speed tests; (**c**) E_1_ and E_2_ from the variable load speed tests.

Strain responses during the RRLS tests of the sample are presented in Figure 12a. Plastic strains appear for the conditioning cycle, as expected. However, for the subsequent cycles, regardless of the load speed, no discernable increase was recorded. Thus, the behavior can indeed be considered purely viscoelastic. The plastic strain from the conditioning cycle has been eliminated from further analysis.

The loading phases are extracted in Figure 12b. Stiffening is apparent with the increase in load speed, leading to the conclusion that it influences the material response. Further analysis is conducted by calculating E_1_ and E_2_ for all cycles, using the same method described in the previous sections (Figure 12c). E_1_ tends to increase in value up to an asymptote at 100 mm/min load speed. The increase is 8%, from 31.79 GPa at 0.1 mm/min, to 34.33 GPa at 200 mm/min. In the case of E_2_, an increase of 3.46% has been identified from 26.23 GPa at 0.1 mm/min, to 27.14 GPa at 200 mm/min. Since both modules tend to rise with load speed, but do not converge to the same value, the bi-linear elastic behavior cannot be explained solely by viscous effects, but rather by the reversible reorganization of the load bearing constituents of the fiber—the microfibrils.

### 3.3. Mechanical Response during Creep

**Figure 13:**
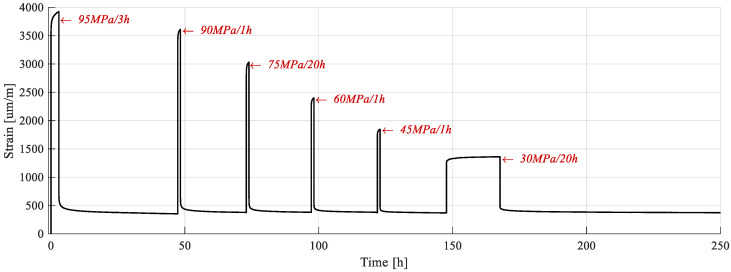
Deformation response during a creep-recovery test on a conditioned sample.

The strain response of the sample subjected to cyclic creep-recovery with regressive load is presented in Figure 13. During all creep phases, a viscous response was apparent, as deformation tended to increase. The same was noted during the recovery phase, when strain decreased over time towards stabilization.

The sample only partially recovered during the conditioning cycle, and residual strains were recorded at the end. For the subsequent cycles, all strain accumulated during creep fully recovered. This demonstrates that plastic deformations formed only during the conditioning stage, and that the behavior of the material was viscoelastic thereafter.

In all subsequent analyses of the data presented in this section, the conditioning cycle was not considered, and the plastic strain resulting during this cycle was subtracted from those that followed.

**Figure 14:**
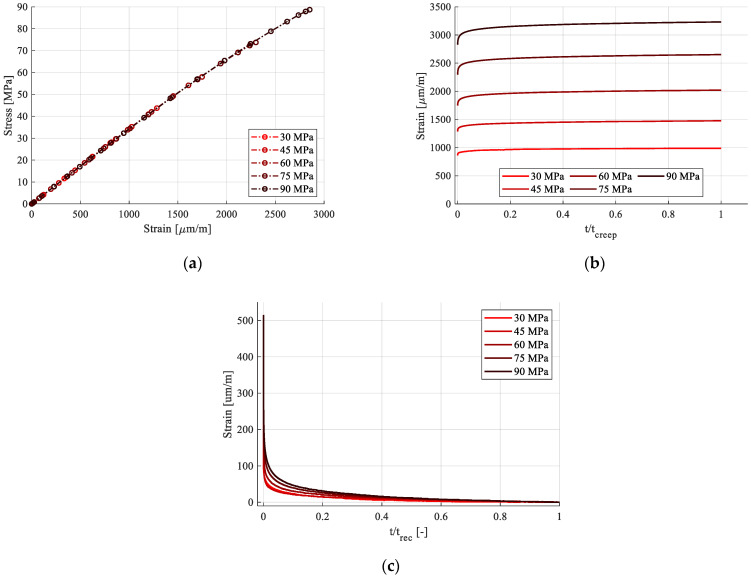
Loading phases of the creep-recovery cycles: (**a**) loading; (**b**) creep; (**c**) recovery.

The loading phases of the cycles are extracted and represented in Figure 14a. They are superposed, proving that no mechanical property degradation has occurred due to the maintaining of load. Since each cycle′s stress level varies, only E_1_ can be utilized for comparison. It has been calculated in the 100–1000 µm/m strain range and is presented, for all cycles, in Table 5.

The creep phase is extracted in Figure 14b and the recovery phase in Figure 14c. Both the creep and the recovery times have been normalized by dividing them with the total creep/recovery duration for each phase. An increase in viscoelastic response can be seen in both phases with the increase in stress level. During recovery, strain dissipates completely, thus clarifying that no plastic strain is accumulated, and that all time variable strain is viscoelastic.

The fact that no plastic strain is formed after the conditioning cycle, corroborated by the findings presented in the previous sections, suggest that the threshold for plastic strain cumulation was shifted. Since the subsequent cycle is close to the conditioning cycle, it can be stated that the shift is to the highest stress level in the load history.

**Figure 15:**
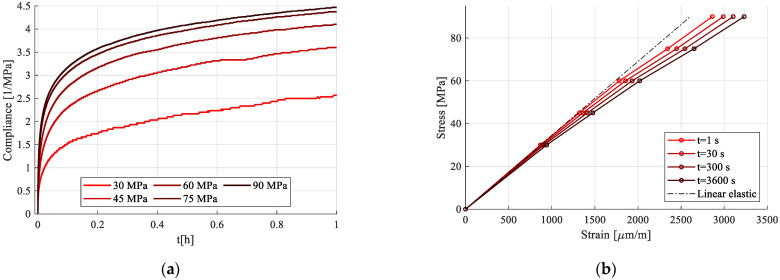
Creep-recovery tests analysis: (**a**) compliance curves of the creep phases; (**b**) isochronous curves.

When plotting the compliance curves for each creep phase (Figure 15a) obtained by dividing the strain response in creep by the stress (Equation (1)), nonlinearity became apparent. Figure 15a contains the transient compliance for only the first hour of creep for all load cycles. This pointed to the fact that viscoelastic strain did, indeed, increase with stress, but not proportionally. In other words, it was nonlinear with respect to stress. The nonlinearity was further visible when extracting isochronous curves in stress–strain coordinates, Figure 15b.

(1) $$D_{\left(t\right)}=\frac{\varepsilon_{\left(t\right)}-\varepsilon_{0}}{\sigma },$$

with $D_{\left(t\right)}$ representing the transient compliance during creep, $\varepsilon_{\left(t\right)}$ as the transient strain during creep, $\varepsilon_{0}$ as the strain produced during loading, and σ representing creep stress.

Data recorded for strain during loading and during creep is extracted in Table 5. Data was processed by calculating the total strain recorded during a creep-recovery cycle, $\Delta \varepsilon_{tot}$, and, of it, the ratio of transient strain, $R_{V}$, Equation (2).

(2) $$R_{V}=\frac{\Delta \varepsilon_{c}}{\varepsilon_{0}+\Delta \varepsilon_{c}}*100,$$

The ratio of transient response, which is only viscoelastic for this dataset, tends to remain constant. This leads to the conclusion that the nonlinearity exhibited in the instantaneous component was inherited by the viscoelastic component as well.

**Figure 16:**
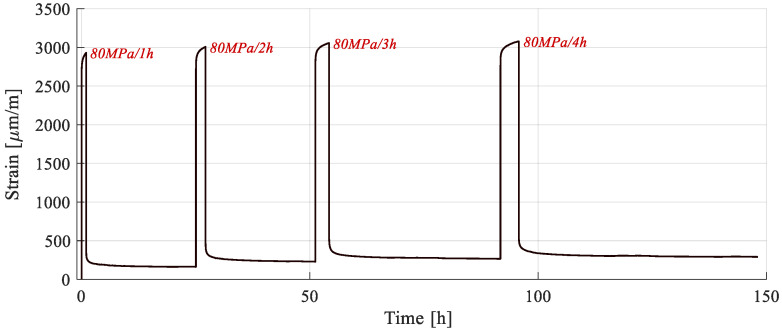
Strain response during a creep-recovery test.

The response of the sample subjected to cyclic creep-recovery with progressive creep duration is presented in Figure 16. While viscous response was present, both during creep and recovery, the sample never fully recovered. Since the load was kept constant, the only variable was creep duration, leading to the inference that it influenced the plastic strain, which increased after each cycle. This time-dependency points to the fact that the permanent strains are viscoplastic. This viscoplastic component is attributed to the irreversible reorganization of the amorphous phases in the composite structure [33].

**Figure 17:**
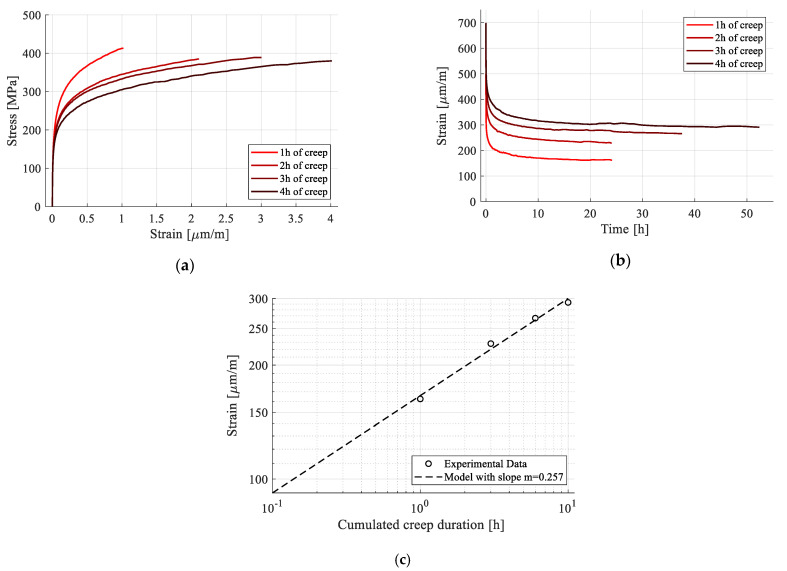
Analysis of the strain response during creep-recovery tests: (**a**) creep phases; (**b**) recovery phases; (**c**) plastic strain with respect to cumulated creep duration, in logarithmic time scale.

Further analysis was conducted by extracting the recovery phases (Figure 17b). As for the previous procedure, recovery was considered to be achieved when the strain had a variation of less than 10 µm/m during ten consecutive hours. An increasing period was required for stabilization, due to the increase in creep duration and viscous strain formation. As for the viscoplastic strain, it is evident that, while increasing with creep duration, the strain rate tends to decrease (Table 6). This effect was also observed during the creep phases (Figure 17a), in which the total transient strain decreased due to the same viscoplastic component. Therefore, even though the creep duration increased from one cycle to the next, the ratio of viscous strain to elastic strain, computed in Table 6, remains close to 14%.

The cumulated viscoplastic strain was extracted as the average of strain data for the last hour of recovery (Figure 17c). The effect of strain rate decrease is clearly seen on a logarithmic scale, where a linear evolution is present. It is thus inferred that viscoplastic strain follows a power law with respect to creep duration, with a sub unitary exponent, Equation (3).

(3) $$\varepsilon_{pl\left(t\right)}=C*t^{m},$$

where $\varepsilon_{pl\left(t\right)}$ represents the viscoplastic strain, C is a multiplication constant, t is the creep duration, and m is the power law exponent.

For the present experimental dataset, the exponent has been calculated as *m* = 0.257 and the constant *C* = 166.08. The fact that plastic strain follows a linear evolution in a logarithmic scale, with respect to time (including the one produced during loading of the first cycle) shows that all permanent strain, including that of the first loading cycle, can be assimilated into a viscoplastic response.

In terms the percentage of viscous response, calculated with (2), this was higher than in the previously presented creep-recovery tests, as the viscoplastic component was also present, and the creep duration was longer.

In Table 6, the strain data for the different phases of the creep-recovery cycles is extracted. E_1_ was calculated for comparison purposes. No significant difference was observed when compared with the previous types of tests. It also tends to remain constant throughout the testing cycles, with the highest variation occurring between the first and the second cycles. This is attributed to the viscous effects present during loading, which have been revealed in previous tests.

### 3.4. Summary

The characterization procedures presented allowed for the determination of the tensile mechanical properties of the studied FFRP. It has exhibited a bi-linear stress–strain curve, with a yield stress at approximately 20% of UTS and a tensile modulus of approximately 34–35 GPa and 26–29 GPa, before and after yield, respectively. It has been determined that, during loading, both viscoelastic and viscoplastic behaviors can be distinguished, but they cannot alone explain the bi-linear behavior of the material.

The viscoelastic components can account for up to 8% of total strain, dependent on load speed. In creep, this viscoelastic component was shown to be nonlinear with respect to stress, and it can amount to an additional 12% of strain, for load durations of 1h. The plastic strain forms, depending on the stress level after a threshold has been exceeded. In creep, the plastic strain increases with load duration as well, proving its viscoplastic nature. Regarding the threshold, it shifts to the highest reached level, concluded from the tests that included mechanical conditioning.

## 4. Conclusions

Samples fabricated from a unidirectional flax fiber—epoxy resin composite material have been subjected to multiple types of experimental procedures to determine the material′s mechanical response. The laminate lay-up, with the reinforcement oriented in the load direction, allowed for determining the fiber dominant behavior of the composite.

The main conclusions drawn from the presented study are:

- The bi-linear tensile behavior of the material comprises a viscoelastoplastic component.
- The viscoelastic component is nonlinear with respect to stress level.
- Viscoplastic strain appears when a stress threshold is exceeded. This threshold coincides with the yield point for a fresh material, but shifts to the highest stress level when loaded above it.
- The viscoplastic strain follows an exponential evolution with respect to time.

The present study has shown the complex behavior of flax fiber-reinforced composite materials, for which the viscous effects cannot be neglected in structural applications.

When designing load bearing structures, appropriate constitutive equations need to be used for a successful prediction of the material behavior. Examples are the Schapery constitutive equation for viscoplasticity and Zapas–Crissman integral for viscoplasticity. In the case of long fiber reinforced composites, these would require coupling with the laminate theory for orthotropic media. The present data provides a basis for the identification of model parameters for the studied reinforcement direction.

## Figures and Tables

**Table 1:** Parameters for RPL tests.

Cycle No.	Load Speed [kN/min]	Stress [MPa]
1	50	30
2		60
3		80
4		100
5		120
6		150
7		180
8		210
9		240
10	10	270

**Table 2:** Parameters for RRLS tests.

Cycle	Stress [MPa]	Load Speed [mm/min]
Conditioning	120	200
1	100	200
2		100
3		50
4		10
5		5
6		2
7		1
8		0.5
9		0.1

**Table 3:** Calculated mechanical properties of the flax fiber—epoxy resin composite.

Property	Unit	Value
E1	[Gpa]	35.88 ± 0.55 (1.55%)
E2	[Gpa]	26.30 ± 0.75 (2.85%)
$\sigma_{y}$	[Mpa]	58.21 ± 8.16 (14.02%)
$\sigma_{u}$	[Mpa]	259.60 ± 25.43 (9.79%)
$\varepsilon_{u}$	[µm/m]	8906 ± 826 (9.27%)
ν	[-]	0.34

**Table 4:** Calculated E_1_, E_2_, and plastic strain for the repeated progressive load tests.

σ [MPa]	E_1_ [GPa]	E_2_ [GPa]	$\varepsilon_{pl}\;$ [μm/m]
30	35.52 ± 0.41	-	-
60	35.34 ± 0.24	-	17.58 ± 12.27
80	35.09 ± 0.16	-	59.80 ± 31.61
100	35.05 ± 0.25	-	140.12 ± 47.79
120	34.93 ± 0.14	-	238.02 ± 68.83
150	34.90 ± 0.17	-	369.82 ± 75.86
180	35.06 ± 0.17	28.76 ± 0.25	508.41 ± 91.87
210	35.15 ± 0.15	29.04 ± 0.22	647.35 ± 117.09
240	35.20 ± 0.19	29.27 ± 0.26	740.39 ± 108.82
270	34.86 ± 0.27	29.23 ± 0.41	884.03 ± 102.11
300	35.09 ± 0.37	29.44 ± 0.07	-

**Table 5:** Strain data from creep-recovery tests with regressive load.

Creep Stress [MPa]	E_1_ [GPa]	$\varepsilon_{0}$ [µm/m]	$\Delta \varepsilon_{c}$ [µm/m]	$\Delta \varepsilon_{tot}$ [µm/m]	$R_{V}\;\;[\%]$
30	34.57	863	124	987	12.56
45	34.46	1310	184	1494	12.32
60	34.48	1775	254	2029	12.52
75	34.34	2327	328	2655	12.35
90	34.30	2852	402	3254	12.35

**Table 6:** Strain data from creep-recovery tests with progressive creep duration.

Creep Duration [h]	E_1_ [GPa]	$\varepsilon_{0}$ [µm/m]	$\Delta \varepsilon_{c}$ [µm/m]	$R_{V}\;[\%]$	$\Delta \varepsilon_{r}$ [µm/m]	$\Delta \varepsilon_{pl}$ [µm/m]
1	34.40	2494	436	14.88	335	163
2	34.90	2460	384	13.50	376	65
3	34.96	2439	389	13.76	406	38
4	35.19	2432	380	13.51	436	27

## Data Availability

Not applicable.
